# Erythromelanosis follicularis faciei et colli with reticulated hyperpigmentation of the extremities

**DOI:** 10.1002/ccr3.1095

**Published:** 2017-08-16

**Authors:** Fahad Mohammad Al‐Saif, Abdullah Ali Baqays, Hadeel Fahad AlSaif, Ahmed Abdullah Alhumidi

**Affiliations:** ^1^ Department of Dermatology College of Medicine King Saud University Riyadh Saudi Arabia; ^2^ Dermatology Resident Saudi Commission for Health Specialties (SCFHS) Riyadh Saudi Arabia; ^3^ Pediatrics Resident Saudi Commission for Health Specialties (SCFHS) Riyadh Saudi Arabia; ^4^ Pathology Department College of Medicine King Saud University Riyadh Saudi Arabia

**Keywords:** Erythromelanosis, faciei, follicularis, hyperpigmentation, reticulated

## Abstract

Erythromelanosis follicularis faciei et colli (EFFC) is a distinct, rare, and underdiagnosed condition of unknown etiology that is characterized by well‐demarcated erythema, hyperpigmentation, and follicular papules. The pigmentation is usually on both maxillary, preauricular regions, and the cheeks. We report a case of a 12‐year‐old boy with EFFC that was associated with reticulated pigmentation of the extremities.

## Introduction

Erythromelanosis follicularis faciei et colli (EFFC) is a rare disease that was originally described by Kitamura et al. 1. The etiology of this condition is unknown, but an autosomal recessive mode of inheritance, familial cases, and spontaneous mutation have been reported 2, 3. EFFC was initially reported in young male patients, but a recent study showed nearly equal incidence between males and females 4. This disease is characterized by the following triad of symptoms: well‐demarcated erythema of the face and neck, hyperpigmentation, and follicular plugging. We report the case of a patient with EFFC that was associated with unusual acral reticulated hyperpigmentation.

## Case Report

**Figure 1:**
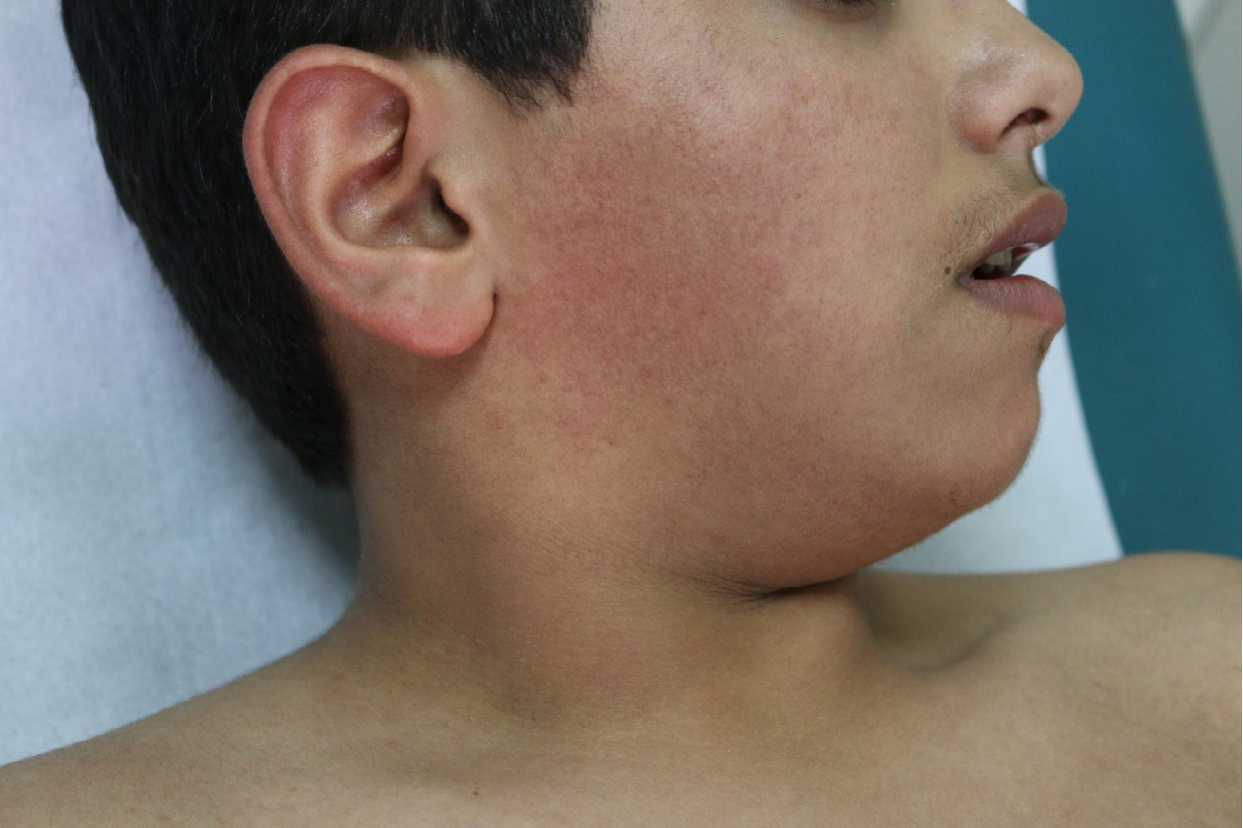
Erythematous patches over preauricular area and cheek.

**Figure 2:**
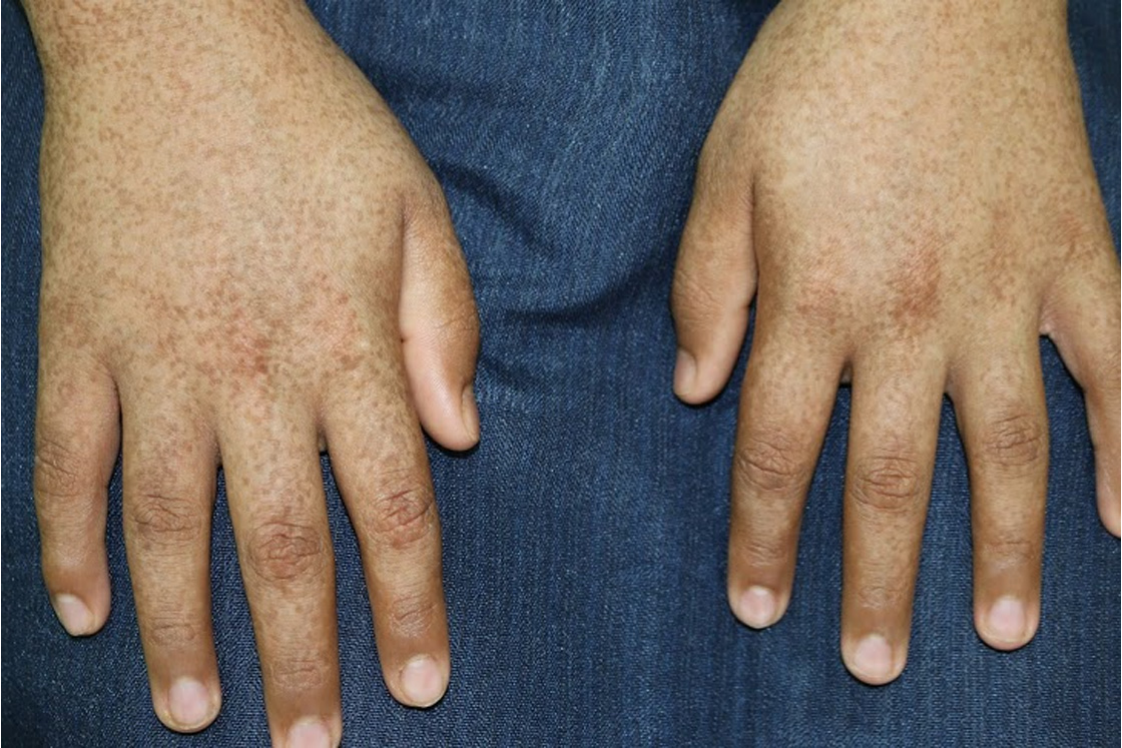
Bilateral reticulated hyperpigmentation over hands.

**Figure 3:**
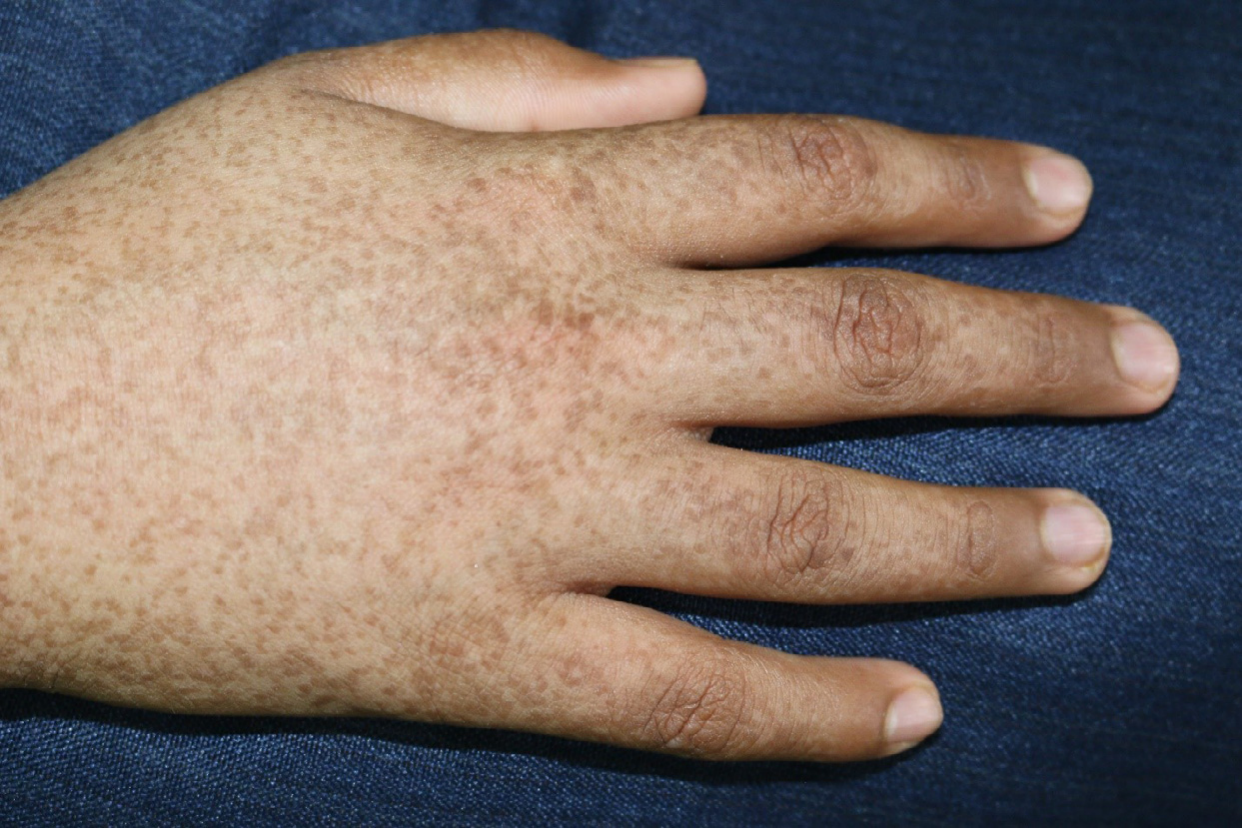
Reticulate hyperpigmented macules present over hands.

**Figure 4:**
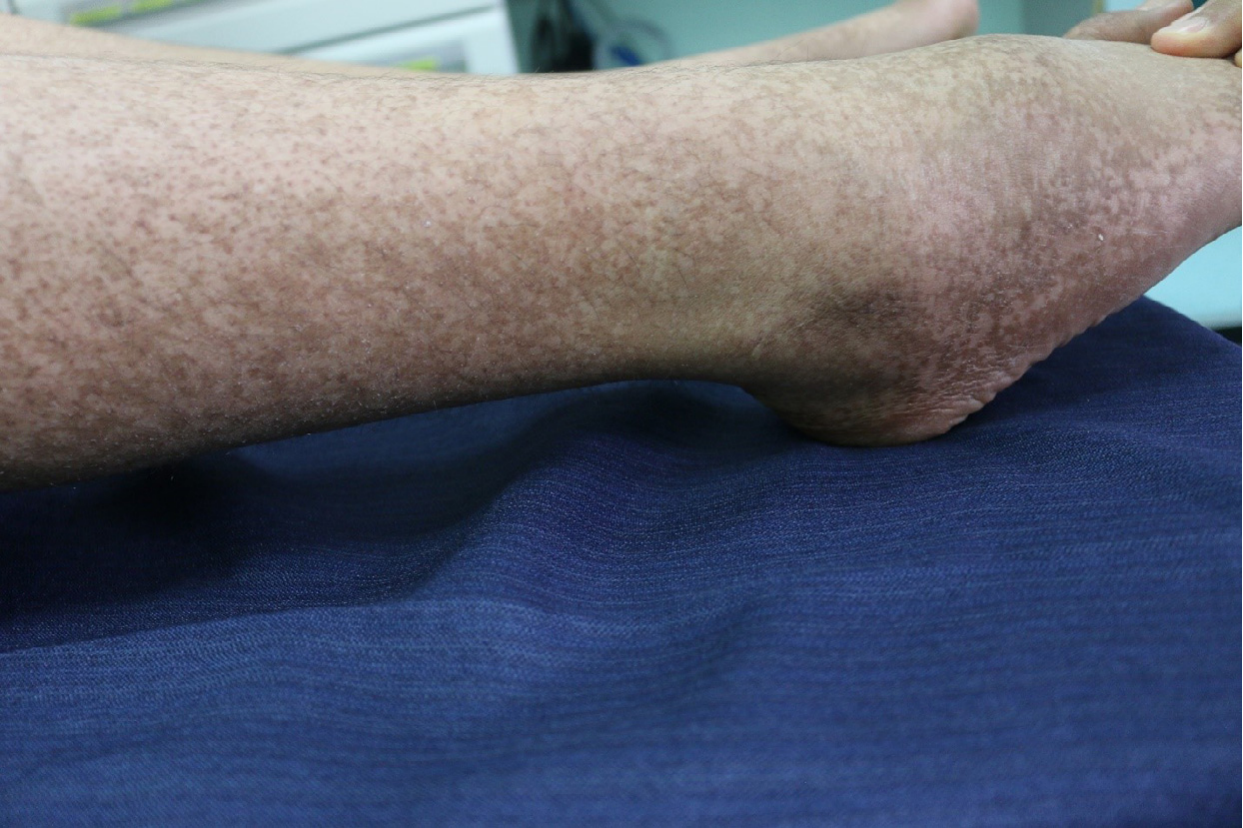
Reticulate hyperpigmented macules present over feet.

**Figure 5:**
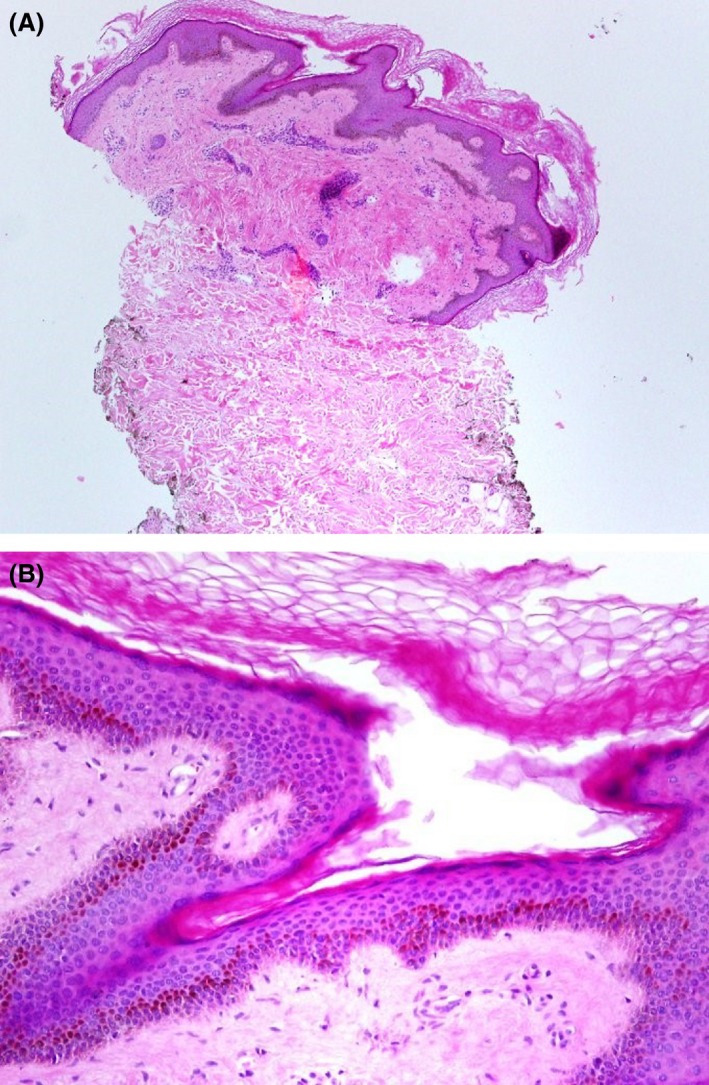
(A) Punch skin biopsy from leg (H/E stain X100). (B) High power (H/E stain X400).

**Figure 6:**
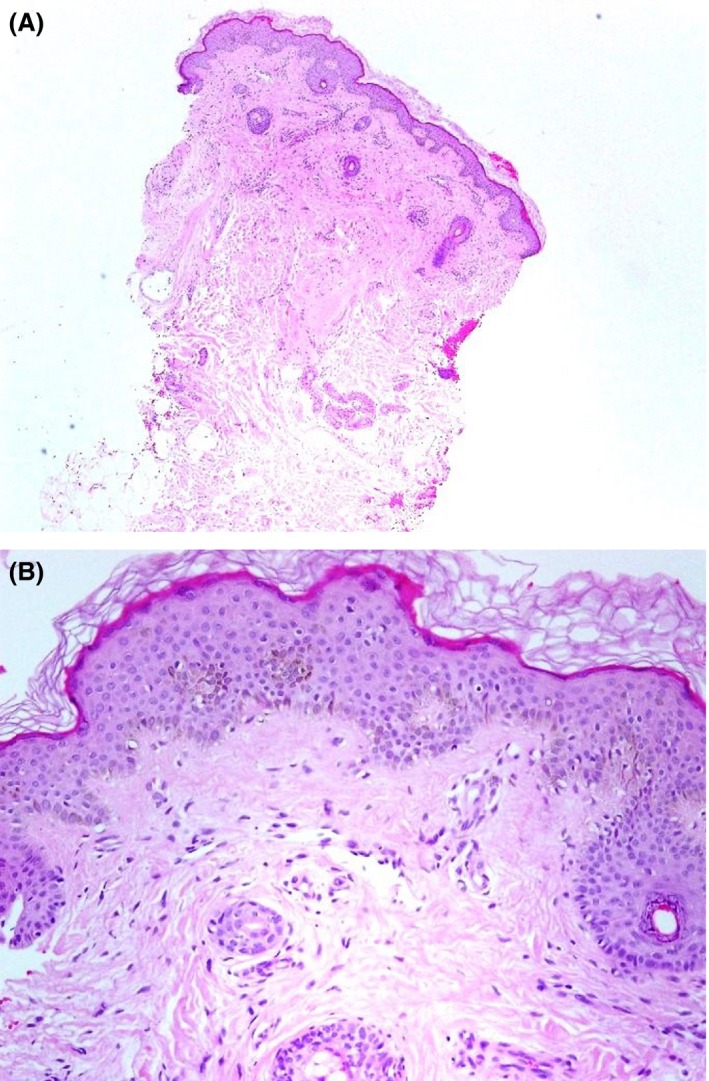
(A) Punch skin biopsy from face (H/E stain X100). (B) High power (H/E stain X400).

A 12‐year‐old boy, born of nonconsanguineous parents after a normal, full‐term vaginal delivery, presented with a 5‐year history of progressive bilateral and symmetrical patches of reddish‐brown pigmentation with tiny papules that begin on preauricular areas and cheeks and gradually spread to the submandibular areas. The child also concurrently developed reticulated pigmentation over the acral part of upper and lower limbs. On examination, there were symmetrical well‐demarcated erythematous patches without telangiectasia, and they were studded with tiny follicular papules over his cheeks. Over both upper and lower limbs, there was diffuse dark‐brown reticulated pigmentation (Figs 1, 2, 3, 4). No palmer pits or hypopigmentations were present. The skin texture of the trunk and limbs was rough with many pale follicular papules. The patient's hair, mucous membranes, and nails were normal, and the results of systemic examination were normal. His family history was negative for EFFC, and the histologic features, although nondiagnostic, correlated well with the clinical features. The microscopic examination of a biopsy taken from the leg skin showed orthokeratosis, follicular keratin plugging, and basal layer hyperpigmentation (Fig. 5A and B). Another skin punch biopsy from the face revealed mild hyperkeratosis, basal layer hyperpigmentation with perifolliculitis, and scattered necrotic keratinocytes in the hair follicle epithelium (Fig. 6A and B).

## Discussion

Erythromelanosis follicularis faciei et colli is a rare disease of unknown etiology, but an autosomal recessive pattern of inheritance and familial cases 2, 3, 4, 5, and spontaneous mutation and chromosomal instability have been reported in some cases 5, 6. This condition was initially reported in young male patients, but a recent study showed nearly equal incidence between males and females 4. The mean age of onset for the disease was 12.1 years 7.

Erythromelanosis follicularis faciei et colli is characterized by well‐demarcated reddish‐brown patches in the preauricular and maxillary areas, and it is associated with keratosis pilaris either over affected areas or over classical sites 3, 4. Telangiectasia can be observed in some patients especially over a sun‐exposed area 8. Pigmentation in EFFC usually occurs in association with erythema and as a result of exposure to sunlight in the predisposed skin 3. Our patient has diffuse dark‐brown reticulated pigmentation over acral areas, and this type of pigmentation has not been previously reported.

Histopathology of EFFC usually reveals follicular plugging, hyperkeratosis, increased pigmentation in the basal membrane, perivascular and periadnexal inflammatory infiltrate, follicular dilatation, and dilated blood vessels in the upper dermis. However, some studies found a correlation between disease severity and basal layer pigmentation in which our case has. 4, 9.

Differential diagnosis includes keratosis pilaris rubra, poikiloderma of Civatte, and Atrophoderma vermiculatum. EFFC has been considered to be a variant of keratosis pilaris in terms of follicular keratin plugging although in skin type I patients keratosis pilaris rubra presents with erythema of the cheeks, but EFFC is associated with well‐defined patches of erythema and pigmentation. Poikiloderma of Civatte is seen in sides of the neck in middle‐aged women. Atrophoderma vermiculatum has honeycombed atrophy that affecting the cheeks, and no erythema or pigmentations are usually noticed 10, 11.

There is no satisfactory treatment for EFFC, and most of the lesions usually recur shortly after treatment is discontinued. Emollients, keratolytic, tacalcitol ointment, metronidazole, hydroquinone 4%, chemical peel, and laser systems have been used with variable results 2, 7, 12, 13, 14.

## Conclusion

We report this case for its rarity and to highlight the association of EFFC with reticulated pigmentation of the extremities.

## Conflict of Interest

None declared.

## Authorship

FMA: conceived the work and reviewed the manuscript. AAB: wrote the manuscript. HFA: wrote the manuscript. AAA: wrote the histopathology section of the manuscript.
